# Successful surgical resection of infected left atrial myxoma in a case complicated with disseminated intravascular coagulation and multiple cerebral infarctions: case report

**DOI:** 10.1186/1749-8090-6-68

**Published:** 2011-05-12

**Authors:** Daisuke Yoshioka, Toshiki Takahashi, Toru Ishizaka, Takuya Higuchi

**Affiliations:** 1 Department of Cardiovascular surgery, Osaka National Hospital, 2-14 Hoenzaka, Chuo-ku, Osaka city, Osaka, 540-0006, Japan

## Abstract

Cardiac myxoma is the most common primary cardiac tumour, but infected cardiac myxoma is relatively rare. Infected cardiac myxoma is very fragile, and has a potential to lead to catastrophic disorder with systemic bacteremia, systemic mycotic embolism, and disseminated intravascular coagulation (DIC).

We present here the successful surgical treatment of a case of infected left atrial myxoma with septic shock, DIC and cerebral infarction without hemorrahage. Collective review of 58 reported cases with infected cardiac myxoma revealed that surgical treatment for it were still challenging and its result was poor. Until date, only one successful surgical treatment for a case complicated by DIC and cerebral infarctions has been reported, and our report describes second such case of successful resection. Even though this report is limited to a case, only aggressive and prompt surgical intervention could relieve the intractable conditions in such a patient with extremely high risk.

## Background

Cardiac myxoma is the most common primary cardiac tumour, but infected cardiac myxoma is relatively rare. To the best of our knowledge, 57 previous cases of infected cardiac myxoma have been reported in the English literature [1-6]. Infected cardiac myxoma almost always causes systemic bacteremia, which easily leads to septic shock, disseminated intravascular coagulation (DIC), multiple organ failure. Infected cardiac myxoma is also very fragile and often occurs with systemic embolism including a cerebral infarction, and hence, surgical resection of the tumor is mandatory for the relief of this intractable condition. However, both DIC and cerebral infarction have a high risk for the open heart surgery with systemic heparinization. In this report, we describe a case of infected left atrial myxoma with DIC and multiple cerebral infarctions, who underwent successful surgical treatment.

## Case Presentation

A 52-year-old man had fever and was diagnosed with an influenza-B virus infection two weeks before admission to our hospital. Despite receiving treatment for the influenza virus, he did not recover and his temperature was elevated at 40°C. A blood culture was positive for gram-positive coccus, and echocardiography showed a large left atrial tumour, which was considered to be atrial myxoma with mobile vegetation. He was referred to our hospital for an emergent operation.

**Figure 1:**
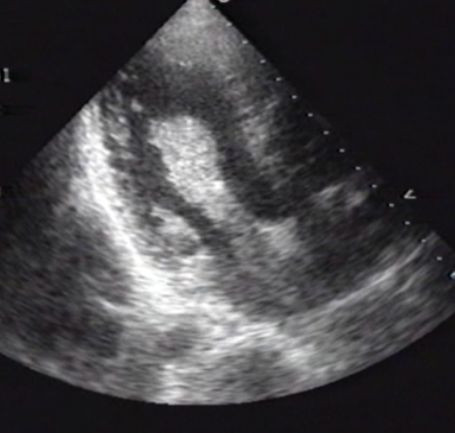
**A large cardiac myxoma (60 × 35 mm in diameter) with a stem attached to the septum of the atrial wall, which prolapsed into the left ventricle during the diastolic phase**.

His physical examination revealed a temperature of 39.5°C and a blood pressure of 80/40 mmHg and a heart rate of 120 beats/min. He had multiple embolic lesions on the distal portion of his extremities. He was delirious and a brain magnetic resonance imaging (MRI) showed multiple small infarctions but fortunately no haemorrhage was detected. Laboratory data revealed a white blood cell count of 13000/mm^3 ^and a C-reactive protein level of 30.0 mg/dl. His platelet count was only 1.0 × 10^4^/mm^3 ^and D-dimer was 12.72 μg/ml, which indicated severe DIC. Echocardiography showed a large mass (60 × 35 mm in diameter) with a stem attached to the septum of the atrial wall, which prolapsed into the left ventricle during the diastolic phase with trivial mitral regurgitation (Figure 1).

**Figure 2:**
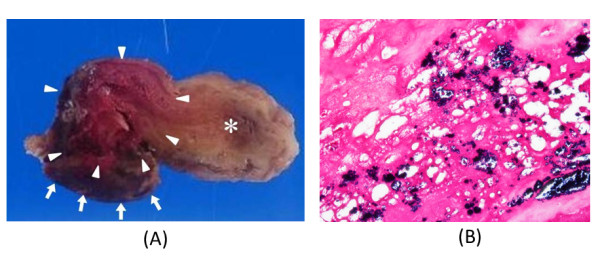
(A): The gross pathological findings were a very fragile myxoid tumor (allow head) attached the septal wall (allow) with the red thrombus and vegetation (*). (B): Hematoxylin and eosin (HE) and showed that the mass was an atrial myxoma, and gram staining of the infected portion revealed the presence of gram-positive coccal bacteria.

An urgent operation was performed using tepid hypothermic cardiopulmonary bypass (CPB) and the usual dose of systemic heparinization. The tumor was completely excised with the attached atrial septum via a trans-septal approach. A small amount of vegetation was observed in the posterior mitral chordae, which was carefully excised without injuring the mitral structure. The gross pathological findings were a very fragile myxoid tumor with the red thrombus (Figure 2A). The platelet concentrate and fresh frozen plasma were transfused after the end of CPB and complete hemostasis was achieved.

After the operation, tumor and blood cultures were positive for methicillin-sensitive *Staphylococcus aureus*. He was still in septic septic shock soon after the operation, but after intravenous immunogloburin and intravenous antibiotic therapy with ampicillin, his general condition was getting better and was extubated at the second postoperative day. The antibiotic therapy with ampicillin was totally administered for six weeks. Histological examination showed that the mass was an infected atrial myxoma, and gram staining of the infected portion revealed the presence of gram-positive coccal bacteria (Figure 2B).

**Figure 3:**
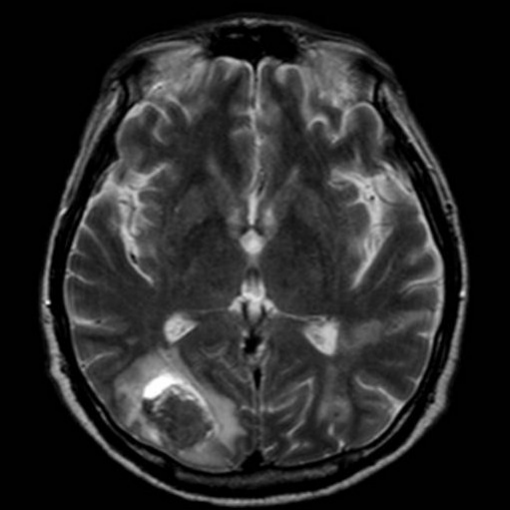
**Post-operative MRI showed a large cerebral haemorrhagic infarction in the right occipital lobe**.

Because of the low platelet count after the operation and to prevent hemorrhagic complications, no anticoagulation therapy was performed in during the post-operative course. But he suffered a brain haemorrhage in the occipital lobe, but fortunately, recovered wit slight cognitive decline (Figure 3). The patient was discharged two months after the operation with a normal C-reactive protein level and no fever was noted. Two years later, he is asymptomatic with no clinical evidence of recurrence.

## Discussion

Although myxoma is the most common benign cardiac tumour, infected cardiac myxoma is very rare. Only 57 English articles on infected cardiac myxoma have been reported thus far in the literature.

The complication of embolism in patients with uninfected atrial myxoma is very common. However, once infected, according to Bough et al., the incidence of cerebral and systemic embolization from infected atrial myxoma is much higher (88% of cases) than those reported from uninfected myxoma (33% of cases) or uncomplicated endocarditis (40% of cases) [7]. In our review, cerebral complications including cerebral infarction, haemorrhage and brain abscess were reported in at least ten cases (16.4%) in the literature, and a systemic embolism including cerebral infarction were reported in at least 24 cases (39.3%). However, actual embolic events may be much higher if brain and systemic computed tomography (CT) scans or brain MRI had been performed preoperatively in all cases. Bacterial cerebral infarction is inclined to be a haemorrhagic infarction, and once cerebral infarction is presented, the perioperative neurological risk is much higher because cardiac surgery requires systemic heparinization for cardiopulmonary bypass. In our case, a post-operative cerebral haemorrhage was presented actually, although a pre-operative cerebral haemorrhage was not detected by brain MRI. Fortunately, the patient recovered with slight cognitive decline. However, if an obvious intracranial haemorrhage is presented preoperatively, cardiac surgery must be postponed and salvage of the patient may be difficult. Collective review of 58 cases with infected cardiac myxoma demonstrated only one case with DIC and cerebral infarction who successful underwent surgical resection of infected myxoma [8].

Including our case, 47 of 58 cases (81.0%) were caused by gram-positive cocci, and 14 of them were caused by *S. aureus*. Five cases combined with DIC have been reported, and all five cases were infected by *S. aureus *[4,5,8,9]. DIC causes a haemostatic disorder and micro-thromboembolisms in small systemic arteries, which leads to multiple organ failure and creates a much higher operative risk. Four of the five patients survived but one died 10 days after surgery as a result of DIC [5].

Our case, which presented with both a bacterial cerebral infarction and DIC, is the second successful case reported. Even though this report is limited to a case, only aggressive and prompt surgical intervention could relieve the intractable conditions in such a patient with extremely high risk.

## Conclusion

In infected cardiac myxoma patients with severe complications, only aggressive and prompt surgical treatment have to be performed for salvage of these patients.

## Consent

The authors confirm that written consent has been obtained from patient in order to publish photographs and relevant clinical information included in the submitted manuscript.

## Competing interests

The authors declare that they have no competing interests.

## Authors' contributions

DY is responsible for acquisition of data and writing the original manuscript. TT, TI and TH are responsible for conception and design as well as critical revision of the manuscript. All authors approved the final version submitted.
